# Assessment of associations between clinical and immune microenvironmental factors and tumor mutation burden in resected nonsmall cell lung cancer by applying machine learning to whole‐slide images

**DOI:** 10.1002/cam4.3107

**Published:** 2020-05-12

**Authors:** Akira Ono, Yukihiro Terada, Takuya Kawata, Masakuni Serizawa, Mitsuhiro Isaka, Takanori Kawabata, Toru Imai, Keita Mori, Koji Muramatsu, Isamu Hayashi, Hirotsugu Kenmotsu, Keiichi Ohshima, Kenichi Urakami, Takeshi Nagashima, Masatoshi Kusuhara, Yasuto Akiyama, Takashi Sugino, Yasuhisa Ohde, Ken Yamaguchi, Toshiaki Takahashi

**Affiliations:** ^1^ Division of Thoracic Oncology Shizuoka Cancer Center Shizuoka Japan; ^2^ Division of Thoracic Surgery Shizuoka Cancer Center Shizuoka Japan; ^3^ Division of Pathology Shizuoka Cancer Center Shizuoka Japan; ^4^ Drug Discovery and Development Division Shizuoka Cancer Center Research Institute Shizuoka Cancer Center Shizuoka Japan; ^5^ Clinical Research Center Shizuoka Cancer Center Shizuoka Japan; ^6^ Department of Clinical Biostatistics Graduate School of Medicine Kyoto University Kyoto Japan; ^7^ Medical Genetics Division Shizuoka Cancer Center Research Institute Shizuoka Cancer Center Shizuoka Japan; ^8^ Cancer Diagnostics Research Division Shizuoka Cancer Center Research Institute Shizuoka Cancer Center Shizuoka Japan; ^9^ SRL Inc Tokyo Japan; ^10^ Region Resources Division Shizuoka Cancer Center Research Institute Shizuoka Cancer Center Shizuoka Japan; ^11^ Immunotherapy Division Shizuoka Cancer Center Research Institute Shizuoka Cancer Center Shizuoka Japan; ^12^ Shizuoka Cancer Center Shizuoka Japan

**Keywords:** CEA, immune microenvironment, machine learning, nonsmall cell lung cancer, tumor mutation burden, whole‐slide imaging

## Abstract

**Background:**

It is unclear whether clinical factors and immune microenvironment (IME) factors are associated with tumor mutation burden (TMB) in patients with nonsmall cell lung cancer (NSCLC).

**Materials and methods:**

We assessed TMB in surgical tumor specimens by performing whole exome sequencing. IME profiles, including PD‐L1 tumor proportion score (TPS), stromal CD8 tumor‐infiltrating lymphocyte (TIL) density, and stromal Foxp3 TIL density, were quantified by digital pathology using a machine learning algorithm. To detect factors associated with TMB, clinical data, and IME factors were assessed by means of a multiple regression model.

**Results:**

We analyzed tumors from 200 of the 246 surgically resected NSCLC patients between September 2014 and September 2015. Patient background: median age (range) 70 years (39‐87); male 37.5%; smoker 27.5%; pathological stage (p‐stage) I/II/III, 63.5/22.5/14.0%; histological type Ad/Sq, 77.0/23.0%; primary tumor location upper/lower, 58.5/41.5%; median PET SUV 7.5 (0.86‐29.8); median serum CEA (sCEA) level 3.4 ng/mL (0.5‐144.3); median serum CYFRA 21‐1 (sCYFRA) level 1.2 ng/mL (1.0‐38.0); median TMB 2.19/ Mb (0.12‐64.38); median PD‐L1 TPS 15.1% (0.09‐77.4); median stromal CD8 TIL density 582.1/mm^2^ (120.0‐4967.6);, and median stromal Foxp3 TIL density 183.7/mm^2^ (6.3‐544.0).

The multiple regression analysis identified three factors associated with higher TMB: smoking status: smoker, increase PET SUV, and sCEA level: >5 ng/mL (*P* < .001, *P* < .001, and *P* = .006, respectively).

**Conclusions:**

The IME factors assessed were not associated with TMB, but our findings showed that, in addition to smoking, PET SUV and sCEA levels may be independent predictors of TMB. TMB and IME factors are independent factors in resected NSCLC.

## Graphical abstract

We were able to quantitatively evaluate the local presence of IME factors that are difficult to evaluate visually, by using a digital pathology platform. The results showed no associations between IME factors and TMB, but significant associations were found with some clinical factors. It is possible that TMB based on whole exome sequencing was not correlated with neoantigen load.

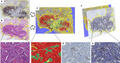

## 1 BACKGROUND

Somatic mutations are presumed to be distributed randomly.^1^ Tobacco smoking has been associated with lung cancer and leads to increased mutation burden.^2^ Nonsmall cell lung cancer (NSCLC), including lung adenocarcinoma and lung squamous cell carcinoma, generally has one of the highest tumor mutation burdens across cancer types.^3^ The mutation burden in lung adenocarcinoma has been reported to be lower in driver‐gene‐alteration‐positive cases than in pan‐negative driver gene cases.^4^ However, smoking status or other clinical factors may have been confounding factors that influenced the association between driver gene alteration status and mutation burden.

Moreover, since tumor mutation burden (TMB) is highly correlated with neoantigens that can be recognized by the immune system, TMB has been expected to serve as a predictive marker for treatment with immune checkpoint inhibitors.^5^

The existence of four different types of immune microenvironment (IME), that is, Type I: TIL+/PD‐L1+, Type II: TIL‐/PD‐L1‐, Type III: TIL‐/PD‐L1+, and Type IV: TIL+/PD‐L1‐, has been proposed by Teng MW et al based on the presence or absence of tumor‐infiltrating lymphocytes (TILs) and PD‐L1.^6^ Assessment of IME factors in their study revealed that CD8‐positive TILs (CD8^+^TILs) exerted effector T‐cell function by recognizing neoantigens in both Type I and Type IV tumors, which are called “hot tumors,” and that PD‐L1 and regulatory T cells exhibited immune resistance and immune tolerance mechanisms in Type I and Type IV tumors, respectively. Foxp3 is known to be a master regulatory gene of regulatory T cells.^7^ In this study we evaluated CD8^+^TILs, Foxp3^+^TILs, and PD‐L1 as IME factors.

According to RNA‐sequencing (RNAseq) data, Type II and Type IV predominate in the IME categories in the Japanese lung cancer population,^8^ whereas Type I and Type III predominate in The Cancer Genome Atlas (TGCA) lung cancer population.^9^ Genome‐based immune cell characterization, other than immunohistochemistry (IHC), is not widely performed, and RNAseq data from a mixture of cancer cells cannot be used to evaluate the local presence of TILs because of contamination by surrounding stromal tissues.

Although the results of a previous study showed no correlation between TMB and PD‐L1 expression level in biopsy samples,^10^ TMB is highly correlated with the number of neoantigens that can be recognized by the immune system, and we assume that some relationship exists between TMB and IME.

PD‐L1 expression in tumors is heterogeneous, and the sample used for the assay may not be representative of the tumor as a whole.^11^ It is recommended that whole tissue sections, instead of “hot spots” defined as small areas with increased TILs, be used to evaluate TILs in heterogeneous tumors.^12^ Visual assessment of immunohistochemistry (IHC) findings by pathologists can be influenced by inherent cognitive and visual biases.^13^

The development of whole‐slide imaging (WSI), which allows entire slides to be imaged and permanently stored at high resolution, enables pathologists to navigate a virtual slide on WSI systems in the same way they navigate Google Maps. WSI systems have led to a number of new opportunities not possible in conventional microscopic evaluation, including quantitative IHC analysis, and measurement of immune phenotypes and their relationship to the IME (eg, tumor vs stroma) using artificial intelligence (AI).^14^, ^15^, ^16^

It is unclear which IME factors, if any, quantified by digital pathology in surgical samples by means of a machine learning algorithm, are associated with mutation burden. In this study we investigated associations between clinical and IME factors and TMB and tried to identify practical predictors of TMB in patients with nonsmall cell lung cancer (NSCLC).

## 2 MATERIALS AND METHODS

### 2.1 Patients

Two hundred of the 246 NSCLC patients who underwent surgical resection of lung adenocarcinoma at the Shizuoka Cancer Center between September 2014 and September 2015 were the subjects of this study. We also conducted a retrospective review of the prospectively collected data of 154 patients with adenocarcinoma (Ad) and 46 patients with squamous cell carcinoma (Sq), using the database of patients enrolled in “Project HOPE” (High‐Tech‐Omics‐based Patient Evaluation). Our study is an additional study of project HOPE.^17^ The Shizuoka Cancer Center launched Project HOPE as a new clinical research program in January 2014 to promote personalized medicine. The purpose of the Project HOPE research program is to identify the cancer characteristics of individual patients by using multiomics‐based analyses across all types of tumors, and in the present study we analyzed cases of surgically resected primary NSCLC by using data obtained according to the Project HOPE protocol. Briefly, we assessed the somatic mutation burden in surgical tumor specimens by performing WES with an Ion Torrent proton platform (Thermo Fisher Scientific). We sequenced the whole exome to an average effective coverage of × 123. We estimated tumor purity using whole exome sequencing (WES) data and the previously reported PurBayes method.^18^ Each patient's serum CEA and CYFRA21‐1 concentrations were measured at the time of their first visit to our institution. Blood samples were obtained by venous puncture, and separated sera were stored at −40°C until analyzed. CEA concentrations were measured with an ARCHITECT^®^ kit (Abbott Japan), and CYFRA21‐1 concentrations were measured with a Lumipulse Presto^®^ kit (FUJIREBIO Inc), which employs the chemiluminescent enzyme immunoassay (CLEIA) method. The upper limits of the normal (ULN) range of CEA values and CYFRA21‐1 values were 5 and 3.5 ng/mL, respectively. Project HOPE was conducted in accordance with the “Ethical Guidelines for Human Genome and Genetic Analysis Research in Japan,” which were revised in 2013.^19^ We obtained consent from each of the patients prior to their participation in this study.

### 2.2 Pathologic procedures and immunohistochemistry

We selected formalin‐fixed paraffin‐embedded (FFPE) blocks from surgical specimens of the primary tumor containing the tumor center and invasive margin in each case, which is considered suitable for evaluating TIL. We recognize that there is an ongoing debate in the broader oncological community about whether assessment of the tumor invasive margin might be more relevant for evaluation of the tumor IME than evaluation of the tumor and stroma separately. In this study, we assessed the density of stromal TILs relative to density of tumor TILs, recommended by an International TILs Working Group^12^ as a factor representing the tumor IME. Representative samples were serially cut into 3‐μm sections and mounted on glass slides. Staining of the sections was performed in the following order: hematoxylin and eosin (H&E), PD‐L1, AE1/3, CD8, and Foxp3.

The sections were incubated at room temperature with primary antibodies against PD‐L1 28‐8 (Abcam[ab205921]) in a 1:200 dilution for 60 minutes, CD8 (Abcam[ab4055]) in a 1:2000 dilution for 60 minutes, and Foxp3 (Abcam[ab20034]) in a 1:200 dilution for 60 minutes, then incubated with a postprimary antibody for 30 minutes and a polymer for 30 minutes according to the manufacturer's recommendations. The sections stained for AE1/AE3 were incubated at room temperature with primary antibodies against AE1/AE3 (DAKO[IR053]) in a 1× (ready to use) for 15 minutes, then incubated with a postprimary antibody for 8 minutes, and finally with a polymer for 8 minutes. All slides were processed on the Autostainer Bond‐III platform (Leica Biosystems) and visualized with a Leica Bond Polymer Refine Detection Kit (DS9800). Deparaffinization, rehydration, and antigen retrieval were performed with Bond Epitope Retrieval Solution 2 [BERS2] (prediluted; pH 9.0) antigen retrieval solution on a Bond‐III Leica automated slide stainer for 20 minutes at 100°C. The specimens were then counterstained with hematoxylin and coverslipped. Each IHC run contained a positive control (tonsil tissue for PD‐L1(28‐8), tonsil tissue for CD8, tonsil tissue for Foxp3, and colon tissue for AE1/AE3). Two PD‐L1 (22C3)‐positive (5%, 80%) specimens in the outsourcing test were used as positive controls for PD‐L1 (28‐8).

### 2.3 IHC evaluation by digital image analysis

**Figure 1:**
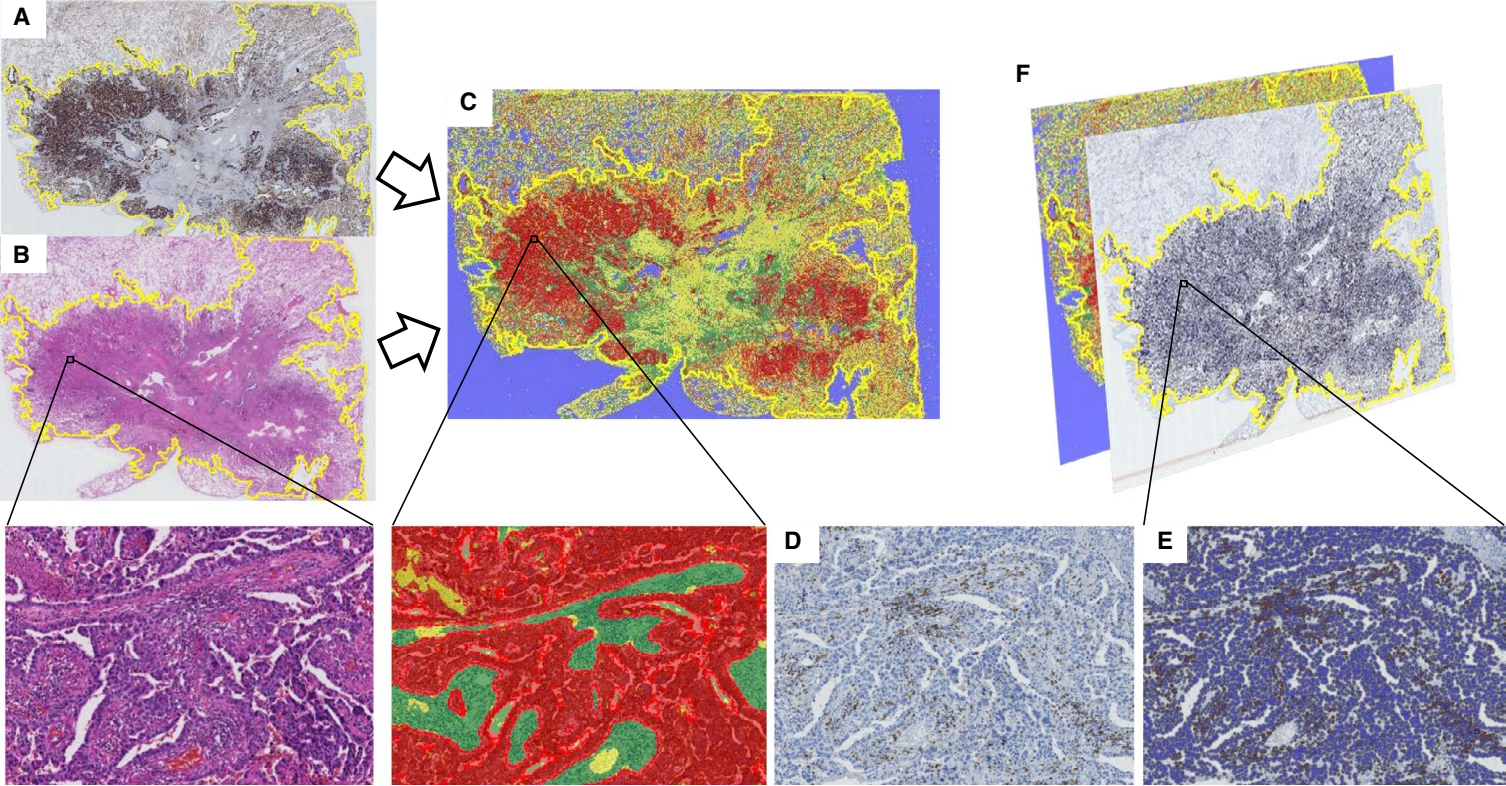
Workflow of quantitative evaluation by digital image analysis using CD8^+^TIL evaluation as an example. Whole‐slide images of 3 μmol/L serially cut tissue sections were stained with AE1/AE3 (A), with H&E (B), and CD8 (D). NSCLC regions (circled in yellow) were annotated for analysis by a pathologist. C, Tissue Classifier: Tumor regions (red), stroma regions (green), and nontumor/nonstroma regions (yellow) were identified using the HALO^TM^ tissue classifier algorithm (a random forest classifier). Pathologists trained the algorithm on AE1/AE3 stained regions set to recognize tumor regions, stroma regions set to stroma regions, and necrotic regions, vessels, inflammation, mucus, anthracosis, and bronchial cartilage regions set to nontumor/nonstroma regions, using a machine learning algorithm. D, Serially cut tissue sections were stained for CD8 (CD8‐positive cells in brown). E, Cell Segmentation: A digital image analysis mark‐up at single‐cell resolution (nuclei in the tumor area and stroma area in blue, CD8‐positive cells in brown). F, HALO^TM^ multiplex IHC v 2.2 machine learning algorithm (a random forest algorithm) can quantitatively evaluate IHC markers in the cytoplasm, nucleus, and/or membrane. This algorithm is run within the annotated region and performs cell segmentation and scoring the TPS of PD‐L1, CD8 cell density in the stroma area, and Foxp3 cell density in the stroma area. Tissue classifier and multiplex IHC analysis can be performed in batch mode

Workflow of quantitative evaluation by digital image analysis in WSI is shown in Figure 1A‐F. All slides were scanned at high resolution on a NanoZoomer Digital Pathology slide scanner (Hamamatsu Photonics), and the digital image analysis was annotated by an experienced pathologist using HALO^TM^ image analysis software v2.2 (Indica Labs). HALO^TM^ is a commercially available machine learning platform, that uses a Random Forest algorithm as a research tool; it has not yet been validated as an in vitro diagnostic. Random Forest algorithms use a decision tree to determine how each pixel in an image should be classified. This algorithm needs to be validated in an independent cohort. In this study, we conducted the validation against manual assessment of the IME factors in a set of 20 randomly selected cases. Tumor regions, stroma regions, and nontumor/nonstroma regions, for example, necrotic regions, vessels, inflammation, mucus, anthracosis, or bronchial cartilage, were identified using the HALO^TM^ tissue classifier algorithm (a random Forest classifier) based on the AE1/AE3 staining pattern. To compensate for nonlinear deformation between tissue sections as much as possible, we evaluated staining on overlaid virtual serial section slides using the HALO^TM^ multiplex IHC v 2.2 machine learning algorithm (a random forest algorithm). Virtual serial section slides stained for PD‐L1, CD8, and Foxp3 were then annotated and quantitatively analyzed using the HALO^TM^ multiplex IHC v 2.2 on a set of 20 randomly selected cases, as previously described by Koelzer VH, et al.^20^ Comparison between conventional and digital assessments of PD‐L1 expression in their study showed a highly significant correlation between pathologist‐based consensus readings and automated PD‐L1 analyses performed using the HALO^TM^ platform (*r* = .97, *P* < .0001). All 20 randomly selected sections were evaluated by two independent pathologists blinded to clinical data. Cells with at least partial linear membranous PD‐L1 staining that reached the threshold decided by the pathologist were annotated as positive. PD‐L1 TPS was quantitatively evaluated by calculating the percentage of positive cells (number of PD‐L1‐positive tumor cells/total number of tumor cells × 100) in the annotated region.

Cells with membranous CD8 staining that reached the threshold decided by the pathologist were annotated as positive. CD8 TILs were quantitatively evaluated by calculating cell density (number of CD8 cells per mm^2^) and percentage (number of CD8 positive cells/total number of cells × 100) in the annotated region. Cells with nuclear Foxp3 staining that reached the threshold decided by pathologist were annotated as positive. Foxp3 TILs were quantitatively evaluated by calculating cell density (number of Foxp3 positive cells per mm^2^) and percentage (number of Foxp3 positive cells/total number of cells × 100) in the annotated region.

### 2.4 Samples

Tumor tissue samples with weights ≧0.1 g were dissected from the surgical specimens together with samples of surrounding normal tissue. The areas from which the tumor samples were dissected were visually assessed as having a tumor content ≧50%. For the DNA analyses, tumor tissue and normal tissue were immediately frozen in liquid nitrogen before DNA extraction. For the RNA analyses, tissue samples were submerged in RNAlater solution (Thermo Fisher Scientific), minced, and stored overnight at 4°C before RNA extraction. Whole blood was collected as a control for WES. We estimated tumor purity using WES data and the previously reported PurBayes method.^17^ Because of a possible correlation between low tumor purity and the false‐negative rate, we excluded from the analysis cases in which tumor purity was less than 20%.

### 2.5 DNA extraction and WES

DNA was extracted from tissue samples using a QIAamp Kit (Qiagen) according to the manufacturer's instructions, and subjected to WES on the Ion Proton System (Thermo Fisher Scientific). WES and variant calling were performed using an Ion Proton AmpliSeq Exome kit and Ion Torrent server as previously reported.^20^ Briefly, 100 ng of DNA was amplified as follows: 99°C for 2 minutes, 95°C for 15 seconds, 10 cycles of 60°C for 16 minutes, and a final hold at 10°C. Incorporated primer sequences were partially digested with FuPa reagent (Thermo Fisher Scientific). Ion Torrent Proton adapters were ligated to the amplicons at 22°C for 30 minutes, then incubated at 72°C for 10 minutes, and the library was purified using Agencourt AMPure XT beads (Thermo Fisher Scientific). The library was quantified by using qPCR, and 7 PM library DNA was sequenced using the Ion Torrent Proton Sequencer with a PI chip V2 according to the manufacturer's protocol (Thermo Fisher Scientific). Torrent Suite software (ver. 4.4) was used to convert raw binary data into sequence reads that were mapped to the reference human genome (hg19 assembly, University of California Santa Cruz Genomics Institute). Somatic mutations were identified by comparing data from the tumor and corresponding blood samples. Single‐nucleotide variants (SNVs) with quality scores < 30, frequency < 10%, or depth of coverage < 20 were discarded. The SNVs of the total exonic mutations for each sequenced tumor included nonsynonymous, synonymous, and indel/frameshift mutations.

### 2.6 RNA extraction and fusion analysis

Total RNA was extracted from approximately 10 mg of minced tissue samples by using the miRNeasy Mini Kit (Qiagen) according to the manufacturer's instructions. Total RNA was assessed using an Agilent 2100 Bioanalyzer (Agilent Technologies). Fusion gene data were analyzed using the Ion Reporter server. The Ion AmpliSeq RNA fusion workflow (Thermo Fisher Scientific) was used to detect fusion transcripts targeted by the HOPE fusion panel.^21^

### 2.7 Statistical methods

To get normality in our TMB data, we used natural logarithmic transformation. The Wilcoxon test was used for comparisons between continuous variables. In correlation analysis, we used Pearson's correlation coefficient (*r*). We conducted univariate and multivariate linear regression analysis to develop our prediction model. Multivariate linear regression was performed with a best subset approach in which variables that were significantly related to transformed TMB in univariate linear regression analysis were included. In this approach, we selected prediction model with minimum AIC. *P* values of less than two‐sided .05 were considered to be indicate statistically significance. Tenfold cross‐validation was used for internal validation of the model. All analyses were implemented by R version 3.5.1 (R Foundation for Statistical Computing, Vienna, Austria).

## 3 RESULTS

**Figure 2:**
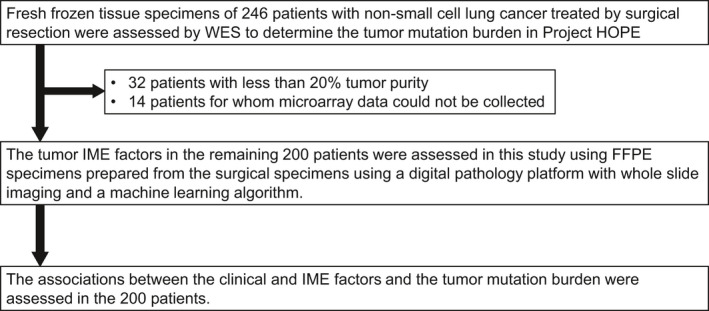
Flow diagram showing the patients included in the analysis

A flow diagram of the patients whose data were included in the analysis is shown in Figure 2. The data of a total of 200 patients who were diagnosed with lung adenocarcinoma or lung squamous carcinoma and underwent surgical resection between September 2014 and September 2015 at the Shizuoka Cancer Center were analyzed. We assessed the somatic mutation burden in fresh frozen tissue specimens by performing WES in project HOPE, and assessed the IME factors through IHC evaluation of FFPE specimens prepared from the same specimens by digital image analysis in our study. The actual PET maximal standardized uptake value (SUV‐max) of seven patients was missing (one patient had not undergone a PET‐CT examination, and six patients were unevaluable). The patient characteristics are listed in Table 1. Median patient age at the time of diagnosis was 70 years (range, 39‐87 years). The patients were predominantly male (63%), smokers (73%), predominantly had adenocarcinoma (77%), right lung primary (58%), upper lobe primary (59%), without actionable mutation (61%) and had pathological stage I disease (63%). Six patients (2.4%) had both EML4‐ALK gene rearrangements and BRAF mutations, and 61 patients (31%) had mutations that conferred sensitivity to EGFR tyrosine kinase inhibitor. The median serum CEA level (range) was 3.4 (0.5‐144.3) ng/mL, median serum CYFRA 21‐1 level (range) 1.2 (1‐38) ng/mL, median exonic mutation burden (range) 2.19 mt/Mb (0.1‐64.3), and median tumor purity 27.2% (20‐99.9).

**Table 1:** Variables associated with mutation burden in univariate/multivariate regression model in log‐transformed TMB scale

Variables	N (%)	Univariate	Multivariate	
		*P* value	*β*‐Coefficient (95% CI)	*P* value
Age				
<70	95 (47)			
≥70	105 (53)	.164		
Gender				
Female	75 (37)			
Male	125 (63)	<.001		
Smoking status				
Never	55 (27)		Reference	
Former/Current	145 (73)	<.001	1.078 (0.759, 1.396)	<.001
Pathological stage				
I	127 (63)			
II, III	73 (37)	.347		
Histological type				
Adenocarcinoma	154 (77)			
Squamous cell carcinoma	46 (23)	<.001		
Primary site				
Right	115 (58)			
Left	85 (42)	.624		
Upper or middle	117 (59)			
Lower	83 (41)	.256		
PET SUV max		<.001	0.056 (0.033, 0.080)	<.001
CEA				
≤5.0 ng/mL	135 (67)		Reference	
>5.0 ng/mL	65 (33)	<.001	0.430 (0.129, 0.731)	.006
CYFRA				
≤3.5 ng/mL	172 (86)			
>3.5 ng/mL	28 (14)	<.001		
Actionable gene alteration				
Presence	77 (39)			
Absence	123 (61)	.004		
CD8				
The number of positive cells in stroma/stroma area (n/mm^2^)		.542		
The number of positive cells in stroma/stroma cells (%)		.910		
The number of positive cells in tumor/tumor area (n/mm^2^)		.894		
The number of positive cells in tumor/tumor cells (%)		.921		
Foxp3				
The number of positive cells in stroma/stroma area (n/mm^2^)		.228		
The number of positive cells in stroma/stroma cells (%)		.206		
The number of positive cells in tumor/tumor area (n/mm^2^)		.673		
The number of positive cells in tumor/tumor cells (%)		.644		
PD‐L1				
The number of positive cells in tumor/tumor cells (%)		.845		

[Correction added on 28 May, after first online publication: In row 1, the value .164 has been moved to 3rd column in this current version.]

Cell density and percentage were significantly correlated with CD8 and Foxp3 in the stroma region and tumor region, respectively (CD8 in stroma: Pearson's *r* = 0.94, CD8 in tumor: *r* = .98; Foxp3 in stroma: *r* = .88, Foxp3 in tumor: *r* = .99), a finding that was consistent with the results of a previous study in melanoma using HALO^TM^ platform.^22^ In this study we mainly assessed the cell density as a representative factor of the tumor IME.

**Figure 3:**
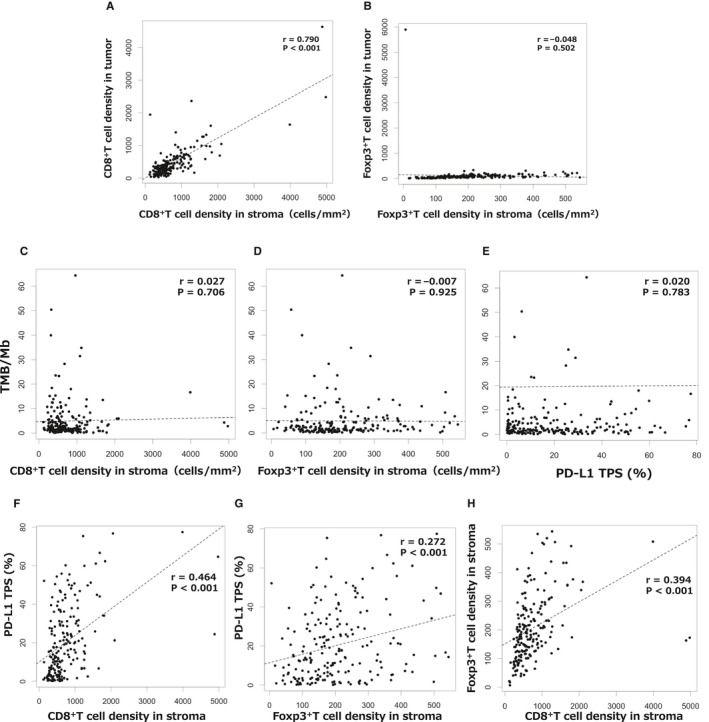
Correlations between CD8^+^ T cell density in the stroma and CD8^+^ T cell density in the tumor (A), Foxp3^+^ T cell density in the stroma and Foxp3^+^ T cell density in the tumor (B), CD8^+^ T cell density in the stroma and PD‐L1 TPS (C), Foxp3^+^ T cell density in the stroma and PD‐L1 TPS (D), PD‐L1 TPS and TMB (E), CD8^+^ T cell density in the stroma and PD‐L1 TPS (F), Foxp3^+^ T cell density in the stroma and PD‐L1 TPS (G), and CD8^+^ T cell density in the stroma and Foxp3^+^ T cell density in the stroma (H). The black dotted line shows the correlation between the data on the horizontal axis and the data on the vertical axis as described by Pearson's correlation coefficient (*r*)

The results of the quantitative evaluations of each IME factor are shown in Table 2 and Figure 3. CD8^+^TIL density and Foxp3^+^TIL density were significantly higher in the stroma area than in the tumor are (*P* < .0001). Median PD‐L1 expression (range) was 15.2% (0.1‐77.5). Although CD8^+^TIL density in the stroma area was strongly correlated with CD8^+^TIL density in the tumor area (*r* = .790, *P* < .001; Figure 3A), Foxp3^+^TIL density in the stroma area was not correlated with Foxp3^+^TIL density in the tumor area (*r* = −.048, *P* = .502; Figure 3B). The median tumor area (mm^2^)/stroma area (mm^2^) ratio was 1.02 (range: 0.11‐8.70).

**Table 2:** Results of the quantitative evaluation of IME factors and PD‐L1

		Median density (range)	Median percentage (range)
CD8	Stroma region	582.0 (119.9‐4876.6)	12.6 (2.7‐87.4)
	Tumor region	336.5 (28.2‐4636.5)	6.1 (0.51‐78.7)
Foxp3	Stroma region	183.7 (6.3‐543.9)	3.1 (0.1‐74.0)
	Tumor region	73.2 (6.4‐5893.6)	1.03 (0.09‐57.3)
PD‐L1	Median TPS (range): 15.2% (0.09‐77.4)		

In the present study no correlation was found between TMB and PD‐L1 expression levels in the surgical samples (*r* = .020, *P* = .783; Figure 3E), the same as reported in a previous study,^10^ and no correlation was found between TMB and either CD8 cell density in the stroma or Foxp3 cell density in the stroma (*r* = .027 *P* = .706, −.007 *P* = .925; Figure 3C,D). However, moderate correlations were found between PD‐L1 expression and CD8^+^TIL density, PD‐L1 expression and Foxp3^+^TIL density, and CD8^+^TIL density and Foxp3^+^TIL density (*r* = 0.464 *P* < .001, *r* = .272 *P* < .001, and *r* = .394 *P* < .001, respectively; Figure 3F‐H).

The exonic mutation burden was significantly higher in men (median: 3.3/Mb, range: 0.2‐64.3, *P* < .0001, Wilcoxon test), patients with squamous histology (median: 5.1, range: 0.6‐18.4, *P* < .0001), smokers (median: 3.2, range: 0.2‐64.3, *P* < .0001), patients without an actionable mutation (median: 3.3, range: 0.1‐50.4, *P* = .0001), patients with a serum CEA level above 5 ng/mL (median: 4.0, range: 0.3‐50.4, *P* < .0001), and patients with a serum CYFRA21‐1 level above 3.5 ng/mL(median: 4.0, range: 0.6‐50.4, *P* = .0005). In a previous study, lung squamous cell carcinomas were found to have a higher mutation burden than lung adenocarcinomas,^23^ and the results of the present study are consistent with that finding (median value 5.1 mutations/Mb vs 1.6 mutations/Mb, *P* < .0001; Wilcoxon's test). Interestingly, the range of mutation burdens in the adenocarcinomas (0.1‐64.4 mutations/Mb) was wider than that in the squamous cell carcinomas (0.6‐18.4 mutations/Mb). The CD8^+^TIL density in the tumor area was significantly higher in the adenocarcinomas, and the Foxp3^+^TIL density in the stromal area was significantly higher in the squamous cell carcinomas, (CD8^+^TIL density in the tumor: *P *= .0018; Foxp3^+^TIL density in the stroma: *P *< .0001). On the other hand, there was no significant difference in the CD8^+^TIL density in the stromal area, Foxp3^+^TIL density in the tumor area, or PD‐L1 expression in the tumor between the adenocarcinomas and squamous cell carcinomas (CD8^+^TIL density in the stromal area: *P *= .84; Foxp3^+^ TIL density in the tumor area: *P *= .09; tumor PD‐L1 expression: *P *= .12). We enrolled 65 patients with NSCLC, including 56 patients with nonsquamous carcinoma (non‐Sq) and 9 patients with squamous cell carcinoma (Sq), who received adjuvant chemotherapy for this study. We assessed the associations of the TMB and IME factors with the postoperative disease‐free survival in the patients with NSCLC who received adjuvant chemotherapy. Multivariate analysis did not reveal any significant associations of the TMB and IME factors, with the disease‐free survival in the NSCLC patients who received adjuvant chemotherapy (data not shown).

The clinical variables identified as being associated with exonic mutation burden in the univariate linear regression were: male gender (*P* < .001), smoker status (*P* < .001), PET SUV‐max (*P* < .001), actionable mutation (*P* = .004), squamous histology (*P* < .001), elevated CEA level (*P* < .001), and elevated CYFRA 21‐1 level (*P* <.001) (Table 1). On the other hand, no associations were found between the IME factors CD8‐positive cell density in the stroma (*P* = .542), Foxp3‐positive cell density in the stroma (*P* = .228), or PD‐L1 expression (*P* = .845) and TMB. These seven clinical variables were included in multivariate linear regression based on the backward stepwise approach (Table 1). It revealed smoking status (yes/no), PET SUV‐max value (continuous), and CEA value (≥5.0 ng/mL) as predictive factors. The prediction model for TMB expresses the relevance of TMB as a function of the three clinical variables as follows (Table 3): TMB ~ 0.581*2.938^(smoking status)* 1.058^(SUV‐max)*1.537^(CEA). We conducted repeated 10‐fold cross‐validation to confirm the internal validity for our statistical model selection. We got the result that a model including smoking status, PET SUV‐max, and serum CEA level were selected in 999 918 cases of 1 000 000 iterations. We think this result implied those three factors are definitely associated with TMB in our study data.

**Table 3:** Result of multivariate regression in original TMB scale

	*β‐*Coefficient	95% CI	*P* value
Intercept	0.581	0.433, 0.779	.000
Smoking Status (Yes)	2.938	2.136, 4.041	<.001
PET SUV‐max	1.058	1.033, 1.084	<.001
CEA (≥5.0)	1.537	1.138, 2.077	.006

## 4 DISCUSSION

By using a digital pathology platform with WSI and a machine learning algorithm to analyze entire surgical specimens in this study, we were able to quantitatively evaluate IME factors that are difficult to evaluate visually. The results showed no associations between IME factors and TMB, but significant associations were found with some clinical factors. Several clinical factors were shown to be confounders that influenced the association between actionable mutations and mutation burden, whereas smoking status, PET SUV‐max, and elevated CEA level were found to be independently and significantly associated with mutation burden in patients with resected NSCLC. We believe that the findings in this study will be useful in building clinical scenarios in the immunotherapy era.

The development of targeted next‐generation sequencing (NGS) and application of an NGS clinical sequencing system such as Foundation CDx could promote precision medicine. In evaluations of TMB, the total number of mutations identified using a targeted NGS has been shown to be strongly correlated with the total exome mutation number.^24^, ^25^ However, WES remains a proven method for analyzing genetic alterations in adequate specimens of NSCLC.^26^, ^27^ Furthermore, since we analyzed TMB in fresh frozen specimens with less DNA damage than in studies that used FFPE blocks, we were able to make highly accurate mutation counts.

In current clinical practice PD‐L1 is semiquantitatively evaluated macroscopically. However, there are difficulties in the quantitative evaluation of PD‐L1 such as heterogeneity,^28^ subjectivity, visual traps (also called optical illusions) which is a limitation of macroscopic evaluation of intensity ^13^, ^29^ and cognitive traps. Also, H&E staining of TILs allows only crude, subjective semiquantitative evaluations.^30^ Tissue microarrays (TMAs) are generally used to semiquantitatively evaluate IME factors. Quantitative evaluations of tumor microenvironment in recent years have employed TMA‐based methods because of the heterogeneity of PD‐L1 expression.^28^ Because evaluation of stroma‐infiltrating lymphocytes in whole tissue sections is recommended when evaluating TILs in breast cancer, evaluation on platforms combining WSI and AI is expected to become a standard tool in the future.^12^ In this study, CD8^+^TIL density in the stroma area was strongly correlated with CD8^+^TIL density in the tumor area. CD8^+^TIL concentrations of the stroma area were significantly higher CD8^+^TIL concentrations of the tumor area. Thus, it is important to evaluate stromal CD8^+^TIL density in NSCLC. Recent advances in digital image analysis technology using a platform that combines WSI technology and AI have enabled quantitative evaluation of an entire tissue profile, which was a limit of evaluation by macroscopic evaluation without going through the process of TMA.^14^, ^15^

Immune scores quantitatively evaluated by WSI have been shown to be prognostic factors even in colorectal cancer.^30^ Clinical phase III trials of immune checkpoint inhibitors as adjuvant chemotherapy for NSCLC are also being conducted (ANVIL; NCT02595944, PEARLS; NCT02504372, Impower010; NCT02486718, IFCT‐1401; NCT02273375), and it is thought that objective, quantitative evaluation of the IME in entire surgical specimens may be necessary as a factor to enable stratification of their effects.

A study in a Western country reported that the median mutation burden based on WES was 6.3 mutations/Mb in lung adenocarcinoma patients and 9.0 mutations/Mb in lung squamous cell carcinoma patients.^23^ An East‐Asian study, however, reported a median of 25 mutations in lung adenocarcinoma patients who had undergone resections.^31^ Since the median mutation burden in NSCLC in the present study was 2.1 mutations/Mb. East‐Asian patients seem to have a lower mutation burden than Western patients. The prevalence of more driver mutations in East‐Asian populations compared with Western populations may be the reason for the ethnic differences in mutation burden, but actionable mutation status was not found to be associated with mutation burden in the present study. PD‐L1 expression in the present study was generally low. It is possible that clone 28‐8 (Abcam) showed a generally lower expression than other PD‐L1 antibodies in NSCLC tumor cells.^32^

Smoking was found to be independently associated with mutation burden in the present study, a finding that was consistent with the results of previous studies.^2^ PET SUV‐max value was also found to be independently associated with mutation burden in the present study. PET SUV‐max in primary lung cancer is related to tumor cell proliferation, prognosis, tumor‐related immunity, and histopathological features of aggressiveness.^33^, ^34^, ^35^ In a recent study, PET SUV‐max showed raw *P* values less than .05 in correlation with mutation burden in patients with lung adenocarcinoma,^36^ although not all of the specimens were obtained by surgical resection (57%).

CEA (carcinoembryonic antigen) is an oncofetal antigen produced during fetal life that disappears after birth. Oncofetal proteins reappear in some cancer patients, indicating that certain genes are reactivated as a result of the cells’ malignant transformation. It is well known that smokers have higher serum CEA levels than nonsmokers do. CEA could serve as an ideal tumor‐associated antigen (TAA), because immunizing cancer patients with TAA is expected to induce effective tumor immunity, not serious autoimmune diseases.^37^ This property of CEA may have influenced its association with mutation burden in this study. Moreover, some reports have mentioned serum CEA as a useful tumor burden marker for early prediction of a response to immune checkpoint inhibitors.^38^, ^39^ The applications for the measurement of CEA levels might be candidates for surrogate markers for the serial assessment of mutation burden.

In a recent study, combination ICI treatment was found to provide a survival benefit, over chemotherapy regardless of patients’ tumor mutational burden.^40^ In the present study TMB was not associated with CD8^+^TIL targeting neoantigen. Therefore, it is possible that TMB based on whole exome sequencing was not correlated with neoantigen load. This enigma needs to be addressed in future investigations that evaluate the degree of immunogenicity (eg, poorly immunogenic, highly immunogenic) of each gene mutation.

The present study had several limitations. First, it was performed as a retrospective review of prospectively collected data at a single institution, and the small sample size was relatively small. However, unlike in previous studies conducted at multiple institutions, we think color normalization by autostainer was achieved. Second, we did not define any cut‐off value for mutation burden. Various TMB cut‐off values for predicting the therapeutic effect of immune checkpoint inhibitors have been reported in recent years, but the cut‐off values have not been standardized and are still controversial.^5^, ^10^, ^41^, ^42^, ^43^ Third, not all of the specimens were analyzed in a Clinical Laboratory Improvement Amendments (CLIA) facility, and we could not analyze degree of immunogenicity. Fourth, our study was that we did not compare the outcomes between fresh frozen tissue specimens and FFPE specimens in this study. Although the median insert size and uniformity of sequencing coverage are known to be lower for FFPE specimens than for fresh frozen specimens, use of optimized FFPE samples are reported as a valid alternative source of DNA for whole‐genome sequence cancer diagnostics if fresh frozen specimens are not available.^44^ Fifth, this algorithm needs to be validated in an independent cohort. In this study, we conducted the validation against manual assessment of the IME factors in a set of 20 randomly selected cases. Sixth, WSI analysis algorithms entail some potential artifacts, including segmentation and classification errors. To reduce such errors, pathologists review at each annotation step. Seventh, we have become aware of the following limitations of the machine learning method: (a) Numbers of training regions and training areas are limited; (b) It is hard to assess the size and the shape of the cell nuclei and identify their margins; (c) The training involved in identifying the border between lymphocyte and tumor is difficult; (d) The way of annotation methods of training regions are confined. In order to overcome the above‐mentioned limitations, deep learning methods may be applied to H&E stained WSI for tumor diagnosis, tumor classification, and prediction of actionable gene alteration in NSCLC. WSI analysis using deep learning algorithms in the clinical setting is challenging. Recent studies have revealed the possibility of applying deep learning to imaging analysis.^45^, ^46^, ^47^ The application AI to WSI analysis may reduce turnaround time, lessen heavy workloads, develop more efficient workflows, increase collaboration though multidisciplinary conferences, realize cost savings, and become a tool for educating physicians.^16^ The medical environment will change as we move into an era when physicians have to master AI. We think that the results of the present study shed light on how to evaluate TMB and IME by using deep learning algorithms in a more precise manner, although further validation in another cohort is needed. We propose to conduct a prospective study to evaluate the IME factors quantitatively and predict the genotype (ie, the presence or absence of other actionable mutations) from the phenotype (ie, histological observations) using deep learning algorithms based on training and validation test sets.

In conclusion, no association between IME factors evaluated by WSI analysis using a machine learning method and TMB were found in this study. However, in addition to smoking, serum CEA levels and PET SUV‐max values may be independent predictors of TMB. TMB and IME factors were found to be independent factors in resected NSCLC. This issue should be evaluated and validated in a future prospective study.

## CONFLICT OF INTEREST

The authors declare no conflict of interest directly relevant or directly related to the content of this article.

## AUTHORS’ CONTRIBUTIONS

Akira Ono was involved in study concept and design; acquisition, analysis, or interpretation of data; drafting of the manuscript; full access to all data in the study and responsible for the integrity of the data and accuracy of the data analysis. Yukihiro Terada and Mitsuhiro Isaka were involved in acquisition, analysis, or interpretation of data. Takuya Kawata, Koji Muramatsu, Isamu Hayashi, and Takashi Sugino were involved in pathological support. Masakuni Serizawa, Keiichi Ohshima, Kenichi Urakami, Takeshi Nagashima, Masatoshi Kusuhara, and Yasuto Akiyama were involved in technical, or material support. Takanori Kawabata, Toru Imai, and Keita Mori were involved in study design and statistical analysis. Hirotsugu Kenmotsu, Yasuhisa Ohde, and Toshiaki Takahashi were involved in administrative, technical, or material support. Ken Yamaguchi was involved in study supervision. All authors read and approved the final manuscript.

## ETHICS APPROVAL NUMBER

This study was approved by the Institutional Review Board of the Shizuoka Cancer Center, Japan (Approval No. 29‐65‐30‐1‐3).

## Data Availability

The data from Project HOPE that support the findings of this study are registered with the National Bioscience Database Center (https://humandbs.biosciencedbc.jp/en/) under the accession number hum0127 and will be available from January 1, 2021. The remaining data that support the findings of this study are available from the corresponding author upon reasonable request. The data are not publicly available due to privacy or ethical restrictions.
